# Production and Analytical Aspects of Natural Pigments to Enhance Alternative Meat Product Color

**DOI:** 10.3390/foods12061281

**Published:** 2023-03-17

**Authors:** Allah Bakhsh, Changjun Cho, Kei Anne Baritugo, Bosung Kim, Qamar Ullah, Attaur Rahman, Sungkwon Park

**Affiliations:** 1 Department of Food Science and Biotechnology, College of Life Science, Sejong University, Seoul 05006, Republic of Korea; 2 Livestock and Dairy Development Department (Research), Peshawar 25000, Khyber Pakhtunkhwa, Pakistan; 3 Department of Medicine and Therapeutics, Faculty of Medicine, The Chinese University of Hong Kong, Hong Kong SAR, China

**Keywords:** natural pigments, chlorophyll, anthocyanin, leghemoglobin, plant-based meat

## Abstract

Color is a major feature that strongly influences the consumer’s perception, selection, and acceptance of various foods. An improved understanding regarding bio-safety protocols, health welfare, and the nutritional importance of food colorants has shifted the attention of the scientific community toward natural pigments to replace their toxic synthetic counterparts. However, owing to safety and toxicity concerns, incorporating natural colorants directly from viable sources into plant-based meat (PBM) has many limitations. Nonetheless, over time, safe and cheap extraction techniques have been developed to extract the purified form of coloring agents from raw materials to be incorporated into PBM products. Subsequently, extracted anthocyanin has displayed compounds like Delphinidin-3-mono glucoside (D3G) at 3.1 min and Petunidin-3-mono glucoside (P3G) at 5.1 277, 515, and 546 nm at chromatographic lambda. Fe-pheophytin was successfully generated from chlorophyll through the ion exchange method. Likewise, the optical density (OD) of synthesized leghemoglobin (LegH) indicated that pBHA bacteria grow more rigorously containing ampicillin with a dilution factor of 10 after 1 h of inoculation. The potential LegH sequence was identified at 2500 bp through gel electrophoresis. The color coordinates and absorbance level of natural pigments showed significant differences (*p* < 0.05) with the control. The development of coloring agents originating from natural sources for PBM can be considered advantageous compared to animal myoglobin in terms of health and functionality. Therefore, the purpose of this study was to produce natural coloring agents for PBM by extracting and developing chlorophyll from spinach, extracting anthocyanins from black beans, and inserting recombinant plasmids into microorganisms to produce LegH.

## 1. Introduction

PBM are food products generated from plant-based ingredients and designed to resemble conventional meat in terms of taste, texture and apperance, without the integration of animal components [1,2]. The goal of PBM is to provide a meat-like experience for those who want to reduce their consumption of animal products for ethical, health, or environmental reasons [3]. Some popular examples of PBM include burgers, sausages, and chicken nuggets produced from ingredients such as soy, pea protein, and mushrooms [4,5]. Nevertheless, the widening gap between the existing supply of meat and its imminent demand has increased the need to produce PBM analogs [6,7]. Therefore, demand for PBM products to replace conventional meat is increasing due to issues linked with extensive agriculture including livestock production, animal welfare, and potential climate change have made it unsustainable to increase meat production to meet future demand. Moreover, previous reports suggest that replacing animal protein with plant-based protein in the daily diet reduces the risk of cardiovascular complications, stroke, and type 2 diabetes [8,9,10,11]. Hence, developing better plant-based diets would address the current protein crisis and positively impact the planet and human health [10,12].

Despite ongoing technical developments, the appearance, flavor, taste, and texture of PBM differ from those of traditional meat products. The most critical feature of PBM anlalogs to be mimicked and resemble traditional meat is visible appearance, particularly color attributes [6,11,12]. Consequently, the integration of natural pigments into PBM products has been developed due to deleterious agents associated with their synthetic counterparts [13,14]. Natural pigments cannot be used directly from renewable sources, and incorporating raw materials as coloring agents in PBM has many limitations [15]. These limitations include the unstable nature of natural pigments during high-pressure and temperature processing, low bioavailability, chemical degradation due to oxidation, and deprivation of quality characteristics during storage, and preservation [16,17,18]. However, the addition of natural pigments has remarkable health benefits and strongly promotes the technological and sensory profile of PBM products [6,13,19].

Anthocyanins constitute the most important group of water-soluble natural pigments. Anthocyanins are reddish-purple color and flavonoids that exist in various plants, fruits, flowers, stems, and leaves [20,21]. The daily intake of anthocyanins is estimated to be 180–215 mg per day per person [22,23]. Nonetheless, the Joint Food and Agriculture Organization (FAO)/World Health Organization (WHO) Expert Committee on Food Additives (JECFA) established that anthocyanin-containing extracts had very low toxicity [24]. The fundamental structure of anthocyanins is a flavan nucleus attached to two aromatic rings, including benzopyrylium and phenolic rings, connected by glucose at carbon atom NO. 3 of benzopyrylium [13]. The structural variation of this ring structure comes from various patterns of substitution in the B ring with OH and OCH_3_ groups, different sugar substituents at the 3 and 5 positions, and the potential acylation of sugar substituents with cinnamic and aliphatic acids. Furthermore, anthocyanin-based compounds are susceptible to heat and light, and their degradation causes the conversion of anthocyanins into polymeric pigments. Additionally, the structural changes in anthocyanins are closely linked to pH variations, which ultimately cause color changes [25,26]. Additionally, anthocyanins as natural pigments possess remarkable anti-inflammatory, potent antioxidant, and anticarcinogenic properties [22,27,28].

Similarly, chlorophyll is a pigment abundant in green vegetables and green plants, and plays an important role in photosynthesis [21]. Moreover, chlorophyll- and chlorophyll-related compounds have protective and anticancer effects [29]. Chlorophyll has an Mg^2+^ ion in the middle of the porphyrin ring structure that plays a role in the coloring and energy-absorption processes [30]. Zn-chlorophyll, in which Mg ions are substituted with Zn ions to stabilize color, has also been developed [31]. Moreover, depending on high temperature and oxygen presence, chlorophyll is converted into several derivatives, such as pheophytins, chlorophyllides, and phephorbides. The formation of pheophytin and other derivatives of chlorophyll is irreversible; however, pheophytin is treated with Cu and Zn ions to form a more attractive and stable residue [13]. Likewise, myoglobin, a meat pigment, also has a ring structure with a Fe ion in the middle of the ring structure. The ring structures of chlorophyll and myoglobin are similar, except for the differing central ions. To mimic the structure and chemical properties of myoglobin, the current study focused on replacing Mg with Fe using an ion exchange process [29].

LegH is a pigment present in the roots of legumes that is structurally similar to myoglobin [3,6]. The gene encoding soy LegH was inserted into the genome of the yeast *Pichia pastoris*, enabling the production of high levels of soy LegH. The corresponding evidence suggested that the whole protein fraction of this LegH preparation (Prep) comprises at least 65% LegH, and the remaining residual proteins are from the *Pichia* host. *P. pastoris* is a nontoxigenic and nonpathogenic methylotrophic yeast that has been used in the recombinant expression of both Generally Recognized as Safe (GRAS) and US Food and Drug Administration (FDA)-approved proteins [4,32].

The existing literature regarding the production, extraction and analytical aspects of natural pigments developed for PBM is very limited; however, some corresponding studies have been reported [13,22,29,32,33,34]. Therefore, researchers are taking a profound interest in utilizing natural pigments as replacements for their synthetic counterparts, particularly by developing sustainable extraction techniques for the efficient retrieval of bioactive compounds for use in PBM products and nutraceuticals. To the best of our knowledge, this is the first report describing the extraction and analytical aspects of natural pigments to be incorporated into PBM products.

## 2. Materials and Methods

### 2.1. Chemicals, Biological Agents, and Instruments

#### 2.1.1. Chemicals

The chemicals used in this study were anthocyanins, ethanol, extraction solvent (Bioextrax AB, Lund, Sweden), distilled water, Milli-Q water, chlorophyll, acetic acid, petroleum ether (PE), diethyl ether, silica gel, ferric chloride (FeCl_2_), sodium hydroxide (NaOH), sodium acetate, propanol, agarose, Tris-acetate-EDTA (TAE), methanol, acetonitrile, formic acid, and sodium dodecyl sulfate-polyacrylamide (SDS-PA).

#### 2.1.2. Biological Agents

The biological agents used in this study were black beans, spinach, leghemoglobin (LegH), Luria-Bertani (LB) broth, yeast extract peptone dextrose (YPD) medium, plasmid BHA, plasmid JAN, ampicillin-resistant sequence, and sequences of pBHA, LegH, and pBHA + LegH.

#### 2.1.3. Instruments

The instruments used in this study were a biosafety level 2 cabinet (BSC), CO_2_ incubator, culture dishes, flasks and plates, colony counter, Buchner funnel with No. 1 Whatman filter paper, rotary evaporator, column chromatography assembly, agar plates, spectrophotometer, Milli-Q water purification apparatus, electrophoresis equipment, colorimeter, high-performance liquid chromatography (HPLC), and a 0.45 μm filter.

### 2.2. Chemical Structure and Extraction of Anthocyanins

**Figure 1:**
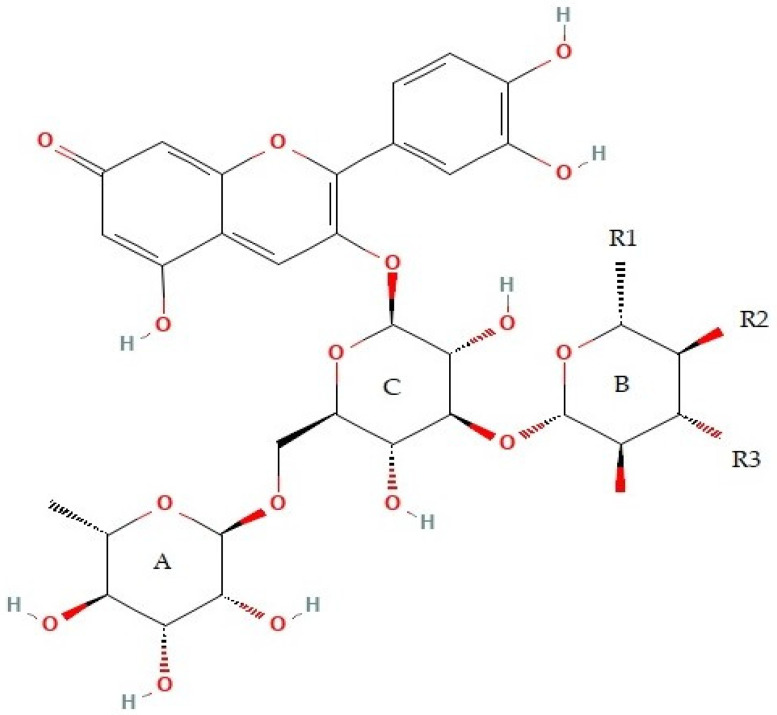
Chemical structure of anthocyanin. R1, R2 and R3 represent the -OH and -OMe groups in various anthocyanidins (PubChem).

Figure 1 shows the chemical structure of anthocyanins. Black beans were purchased from Morning Crops Co. (Gimpo, Republic of Korea), and anthocyanins were extracted from 500 g of black beans using an extraction solvent (Bioextrax AB, Lund, Sweden). The anthocyanins were extracted at 25 °C with acetic acid which was dispensed to a hydroalcoholic solution (60% ethanol) used for extraction to reach a 0.1% acetic acid concentration in the solvent extraction mixture. The resulting mixture was stirred at 250 rpm for 4 h, followed by sedimentation for an additional 1 h. Using vacuum filtration, the supernatant was recovered using Whatman paper No. 1. To eliminate the ethanol, the resultant extract was concentrated using a rotary evaporator by setting the water bath temperature to 50 °C. Subsequently, the concentrated extract was lyophilized, and the resulting lyophilized powder was stored at −80 °C [35].

### 2.3. Chemical Structure and Extraction of Chlorophyll

**Figure 2:**
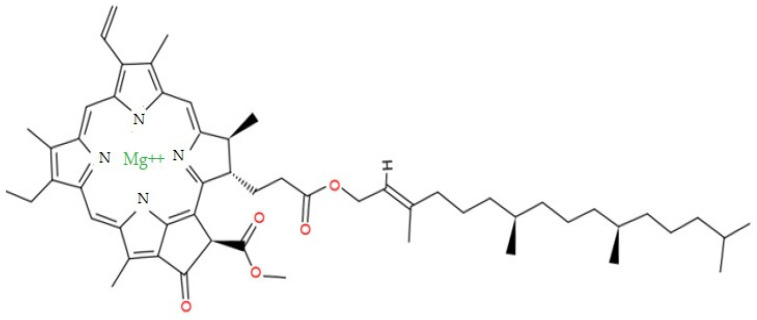
Chemical structure of chlorophyll (PubChem).

The chemical structure of chlorophyll is presented in Figure 2. Spinach (*Spinacia oleracea*) was purchased from Gomgom (Seoul, Republic of Korea). Under controlled conditions, a sample of 500 g was dried at 50–60 °C for 24 h to eliminate moisture. The dehydrated sample, weighing 37.86 g, was extracted with ethanol at a mass: solvent ratio of 1:10 *w*/*v* at 60 °C for 30 min. The crude chlorophyll filtrate was separated using a Buchner funnel with No. 1 Whatman filter paper. A rotary evaporator was used to concentrate the extract. Following concentration, petroleum ether (PE) was added to sonicate and dissolve the extracts. Thereafter, the extracts were stored at −20 °C with no influence of light [36].

#### Synthesis of Fe-Pheophytin by Ion Exchange Chromatography

The process of synthesis of FePhe derivatives has been adopted from Nelson and Ferruzzi [29], and the detection of various FePhe derivatives samples extracted from spinach was separated by ion exchange chromatography through an established procedure of Moustafa and Morsi [37]. Consequently, through the ion exchange method, the extracted chlorophyll sample was added with 1 N NaOH to adjust the pH to 8.5, followed by heating, stirring, and dissolving in distilled water. The sample dissolved in distilled water was adjusted to pH 3 using 3-acetic acids and heated at 60–80 °C for 30 min. A solution of 1.3 M FeCl_2_ and 0.25 M sodium acetate was prepared and dissolved in acetic acid. The prepared acetic acid solution was added five times to the sample, mixed with the heated sample, and further heated at 60–80 °C for 30 min. The pH of the sample was titrated to 8.5 by adding NaOH and distilled water, and then heating and stirring the extract. The sample extracted in distilled water was freeze-dried to form a powder and stored at −80 °C.

**Figure 3:**
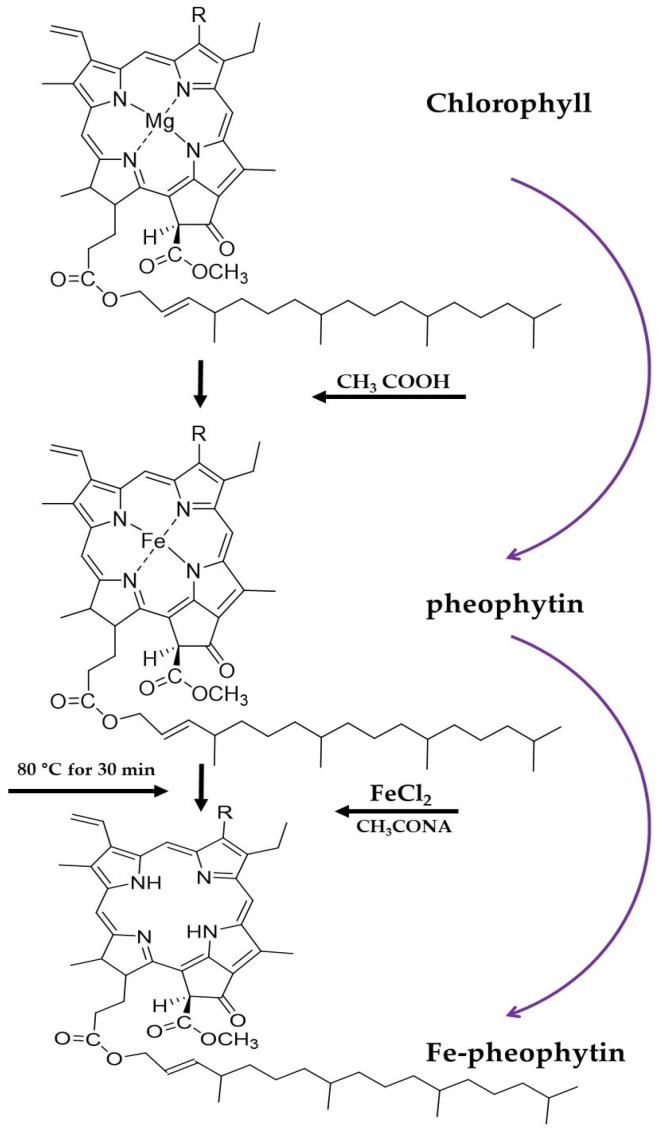
Reaction scheme for the synthesis of pheophytin and Fe-pheophytin. Derivatives of natural chlorophyll are present in crude spinach extract (PubChem).

Subsequently, a column was prepared by mixing silica gel with PE. After filling with silica gel, spinach extract and sand were added to protect the sample layer. PE was added until color separation into green (chlorophyll), yellow (pheophytin), and orange (Fe-pheophytin); when this separation was confirmed, the yellow and orange sample layers were extracted with the addition of PE alongside 10% diethyl ether. The green sample layer was extracted using PE with 5% 2-propanol until the sample was not dissolved in the extraction solvent. After extraction, the sample was stored at −20 °C and protected from light (Figure 3).

### 2.4. Leghemoglobin

#### 2.4.1. Plasmid

The sequence for producing LegH was custom-made by Bioneer Co. (Daejeon, Republic of Korea). This sequence was inserted into the plasmid BHA (pBHA) at a size of 1987 bp [38]. In the case of pBHA cloning, only pBHA recombinant microorganisms can be obtained from an ampicillin-containing medium, as this plasmid confers an antibiotic and ampicillin-resistant sequence. The sequence that produces LegH is 528 bp in size (Bioneer Co.; Daejeon, Republic of Korea) (Table 1).

#### 2.4.2. Microbial Culture

The extraction technology for LegH Prep was adopted from Impossible™ Foods (Redwood City, CA, USA, Private limited) *P. pastoris* production strain, MXY0291 (GRAS notification for soy leghemoglobin protein preparation derived from *Pichia pastoris*) (patent approved, 2017) [39]. This LegH Prep does not contain the production organism or antibiotic resistance genes. Impossible™ Foods has conducted mass spectrometry to identify the *P. pastoris* protein that is present in LegH Prep at >1% of the total protein fraction. The sequence of each protein was analyzed to ensure that the *P. pastoris* proteins present in the LegH Prep did not contain significantly similar homology to known allergens [40].

**Figure 4:**
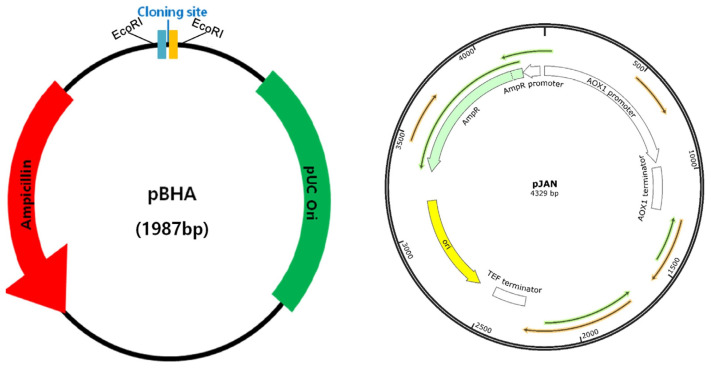
Plasmid (BHA) & Plasmid JAN (Bioneer Co, Daejeon, Republic of Korea).

The production of LegH from legumes using microorganisms was adopted from a previously established procedure described by Jin et al. [41]. The microbial culture was produced using LB broth (Sigma-Aldrich, St. Louis, MO, USA) and LB agar plates (Sigma-Aldrich, St. Louis, MO, USA) as the bacterial culture medium. Ampicillin (Roche Holding AG, Basel, Switzerland) was procured from Sigma-Aldrich (St. Louis, MO, USA). The *Escherichia coli* (DH5a) with or without pBHA (with LegH) were incubated with LB broth in an incubator at 37 C for 24 h. The cultured bacteria were diluted from 10^1^ to 10^9^ and the optical density (OD) was measured at 600 nm every hour for 24 h using a Multiskan SkyHigh Microplate Spectrophotometer (Thermo Fisher Scientific, Waltham, MA, USA). *E. coli* were then separated into media with and without ampicillin, added to an LB plate, and spread to check for growth. Subsequently, pJAN, with an ampicillin resistance sequence, was inserted into *P. pastoris*. Similarly, *P. pastoris* was cultured in yeast extract peptone dextrose (YPD) medium containing 0.1 g/L of ampicillin. The culture process was conducted for up to 48 h at 30 C. Through this methodological process, the generated LegH sequence extracted from pBHA was inserted into pJAN to grow *P. pastoris* (Figure 4). Moreover, pBHA contains a promoter that drives gene expression, a selection marker for identifying cells that have taken up the plasmid, and a multiple cloning site for inserting the gene of interest. The pBHA vector plasmid is named after its components, which include the promoter from the human beta-actin gene (B), the hygromycin selection marker (H), and the polyadenylation signal from the bovine growth hormone gene (A). Similarly, pJAN had a size of 4329 base pairs (bp). The pJAN is a custom-designed or proprietary plasmid developed by a specific research group.

### 2.5. Electrophoresis

The procedure was conducted by mixing 1% agarose gel with 1× TAE buffer (Bioneer, Daejeon, Republic of Korea), and 1× TAE buffer solution. The 50× TAE buffer (Bioneer, Republic of Korea) was diluted 1× with water produced using a Milli-Q water purification system (Millipore, Billerica, MA, USA). A 1 kb ladder from GeneDireX (Taiwan, China) was used. Electrophoresis was conducted at 100 V for 60 min (Mupid-One; Takara, Japan). The sodium dodecyl sulfate–polyacrylamide gel electrophoresis (SDS-PAGE) (Bio-Rad Laboratories, Hercules, CA, USA) method was adopted, as described by Bakhsh, Lee, Bakry, Rathnayake, Son, Kim, Hwang and Joo [19].

### 2.6. Color Measurement

The chromaticity and color coordinates of anthocyanin, Fe-chlorophyll, and myoglobin samples were tested after adjusting the absorbance to 0.7 at 535 nm. Each pigment sample (30 mL) was poured into a 50 mL conical tube (Φ 3 cm). Color indices were measured using a colorimeter (Color Flex EZ colorimeter, Hunter Associates Laboratory Inc., Reston, VA, USA). Before the measurement, a white plate (X = 80.59, Y = 85.72, Z = 91.97, Illuminant D65) was calibrated, and the sample was measured for brightness (L*), redness (a*), and yellowness (b*) using the Hunter scale [42,43,44].

### 2.7. Absorbance Measurement

The absorbance of the anthocyanin, Fe-chlorophyll, LegH, and myoglobin samples was set to a concentration of 0.7% at 535 nm absorbance. Absorbance was measured in the 400–700 nm range using a Multiskan SkyHigh Microplate Spectrophotometer (Thermo Fisher Scientific, Waltham, MA, USA) [45].

### 2.8. High-Performance Liquid Chromatography (HPLC) Analysis

Based on the determination of the standard, the experiment was performed using HPLC under the same conditions. Methanol, water, and acetonitrile (Sigma-Aldrich, Steinheim, Germany) were used as the HPLC-grade products. Samples were prepared by filtration using a 0.45 μm filter (GVS, Bologna, Italy). Agilent 1100 HPLC (Agilent, Santa Clara, CA, USA) and an Agilent C18 column were used for HPLC. For the mobile phase, solvent A was water: ACN: formic acid (45:45:10, vol/vol), and solvent B was 10% formic acid water. The flow rate used was 1 mL/min, the analysis time was 60 min, and the injection volume was 20 μL. The following wavelengths were detected: 277, 515, and 546 nm [46].

### 2.9. Statistical Analysis

Shapiro–Wilk and Levene’s tests were performed to measure the normality and homoscedasticity assumptions. One-way analysis of variance (ANOVA) was used to analyze the data using R Studio (Ver. 4.0.2, Software & Tech Services, Boston, MA, USA). Duncan’s multiple range tests were performed to assess the differences between means. Data are expressed as mean ± standard deviation. Significant differences among the groups were determined using a significance level of *p* < 0.05. All experiments were conducted in triplicate.

## 3. Results and Discussion

### 3.1. Anthocyanin

#### High-Performance Liquid Chromatography

Recently, a sustainable methodology based on green extraction technologies has been adopted for the extraction of bioactive compounds from plant sources. Based on the current green extraction technologies, anthocyanin is a plant-specialized water-soluble compound that confers red, violet, and blue colors to various food products [47]. Anthocyanin samples were analyzed by HPLC at 277 and 546 nm using a c18 column. The standard peaks of Del-delphinidin-3-mono glucoside (D3G) and Petunidin-3-mono glucoside (P3G) were observed at 3.1 and 5.1 min, respectively (S1 and S2). This phenomenon has been documented previously that characterized anthocyanin profiles of red table grape cultivars [48].

Previously, Lianza et al. [49] determined that the efficiency of anthocyanin extraction from untreated black beans was better than that of anthocyanin extraction experiments performed after peeling and drying black beans. The possible reason for this observation is that anthocyanin is water soluble; therefore, rehydration may lead to pigment deprivation during the extraction process. Moreover, it has been proven that anthocyanin extraction is positively affected by the incorporation of hydrochloric acid, rather than acetic acid. Additionally, Teixeira et al. [50] have extracted anthocyanin from broken black bean hulls using optimum conditions with a 30:70 (*v*/*v*) ratio of ethanol and citric acid solution 0.1 mol L^−1^, at a flow rate of 4 mL min^−1^ at 60 °C. This study further indicated that Delphinidin-3-glucoside was the primary anthocyanin that has been linked to numerous health benefits, such as antidiabetic and antiradical activities. Furthermore, Pomar, Novo and Masa [48] estimated a typical HPLC chromatogram of anthocyanin at 546 nm. The authors calculated the chemical structure of anthocyanins by hydrolysis and chromatographic analysis. Various peaks were numbered which correspond to mono glucosides of five anthocyanins found in grapes.

In addition, the amount of anthocyanin extracted varied according to the concentration of acid added to the extraction solvent. When hydrochloric, acetic, formic, phosphoric, and citric acids were compared, hydrochloric acid showed the highest yield. The concentration-dependent extraction (0.1, 0.2, and 0.3%) revealed that 0.3% concentrations of these acids resulted in a higher yield of anthocyanin; however, higher or lower concentrations than 0.3% resulted in lower yields [51]. Similarly, based on the current observations, Pappas, Athanasiadis, Palaiogiannis, Poulianiti, Bozinou, Lalas and Makris [34] conducted a proportional evaluation of diverse novel technologies for the extraction of total anthocyanins from freeze-dried saffron tepals using aqueous solutions of citric acid and the lactic acid at various concentrations. Contrastingly, these results showed that no specific configuration was observed for the type of acid or concentration. The authors further clarified that the best performance for anthocyanin extraction and antioxidant capacity was obtained using a stirred-tank extraction with 1% (*w*/*v*) lactic acid solution; this yielded 3.25 g of cyanidin-3-O-glucoside equivalents (CGE)/kg in the dry weight of tepals. For anthocyanins, quantitative analysis has been widely conducted in terms of the total anthocyanin content. In recent years, however, an increasing number of studies report individual anthocyanin concentrations, and influencing factors have also been investigated. The major contribution to the extraction of anthocyanins from natural sources for food health applications is described in detail by previous authors [16,52,53].

However, because the aim of extracting anthocyanin as a pigment is for the manufacture of PBM as a food product, it is necessary to determine the residual amount of acid that is harmful to the human body and conduct a corresponding safety test. Furthermore, additional experiments on the anti-inflammatory and antioxidant effects of anthocyanins are required. Nonetheless, regarding the toxicity of anthocyanins, previous studies have indicated that this compound is widely used as a natural colorant in food and beverages in Europe, Japan, and the United States [22]. Additionally, JECFA concluded that anthocyanin-containing extracts had a very low or negligible level of toxicity upon consumption [24,54].

### 3.2. Chlorophyll and Fe-Pheophytin

Figure 3 describes the conversion of chlorophyll into its derivatives pheophytin and Fe-pheophytin. Heme protein is considered the preferred protein for consumption [29]. However, the consumption of heme protein has several limitations, including cultural, socioeconomic, and environmental issues [55]. To counter these societal complications, an alternative strategy has been adopted by replacing the heme protein with a structurally similar tetrapyrrole. Chlorophyll molecules can be modified by replacing centrally chelated Mg with other metals, such as Fe, to generate different metalloporphyrins [56,57]. Furthermore, lipophilic and hydrophilic derivatives have been produced and applied in the food and pharmaceutical industries; these derivatives include Zn- and Cu-pheophytins, pyropheophytins, and Cu- and Fe-chlorophyllin as food colorants and nutritional supplements [58]. Inconsistent with the current study FURUYA et al. [59] synthesized and purified the Fe-pheophytin from crude spinach extract successfully. Similarly, the degradation of natural chlorophylls to pheophytins occurs instantly in natural plant extracts [60] and thermally treated vegetables [61]. Moreover, the extraction and incorporation of chlorophyll and its derivatives have been demonstrated in detail [62,63]. In the supporting literature, chlorophyll derivatives, pheophytins, and Zn-pheophytins from chlorophylls were extracted from spinach, characterized, and evaluated for their antioxidant and anti-inflammatory activities [63,64].

Additionally, Nelson and Ferruzzi [29] utilized gastric and small intestine digestion simulation trials with Fe-chlorophyll; overall, this study indicated that the recovery rate of Fe-chlorophyll was 52.3%, the recovery rate of Fe-chlorophyll derivative was 58.7%, and digestion results were stable. Therefore, the development of chlorophyll-derived heme mimetics offers opportunities to expand the current Fe fortification strategies. Previous studies have indicated that the chlorophyll molecule can be modified by replacing centrally chelated Mg with other metals, such as Cu, Zn, and Fe, to form new metalloporphyrin complexes [56,57,58]. A successful trial was conducted for assessing the quantitative yield of chlorophylls into pheophytins and Fepheophytins, supporting these findings, and FePhe derivatives were synthesized at 78.5 ± 0.1% efficiency [29]. Due to the limited available literature, further discussion on this area of research is difficult.

### 3.3. Leghaemoglobin

#### 3.3.1. Microbial Culture

**Figure 5:**
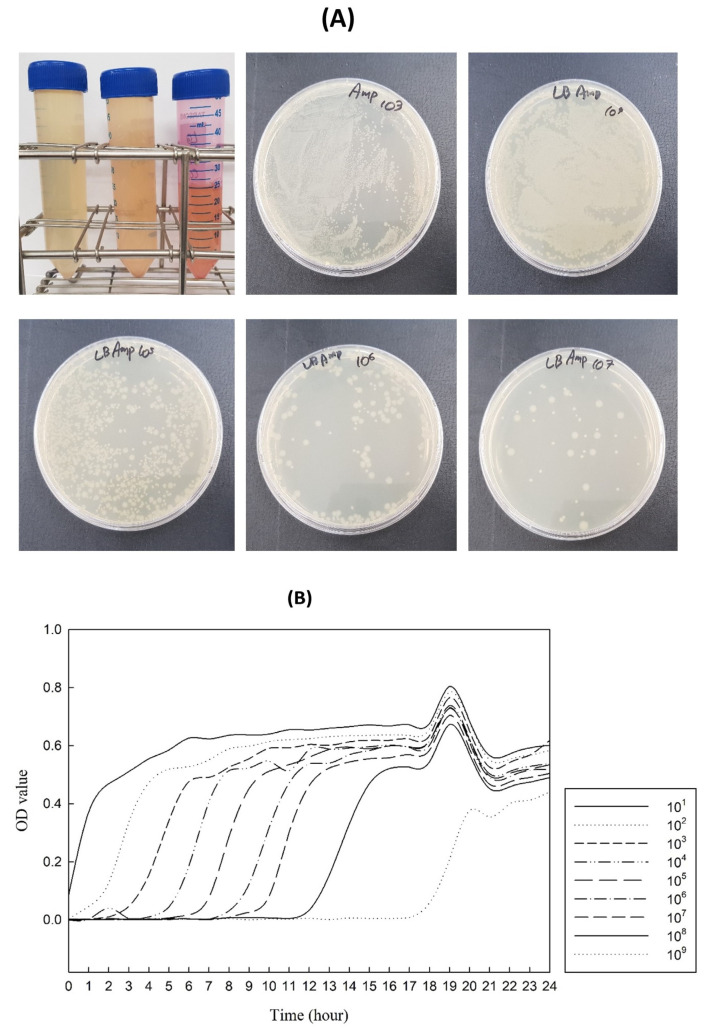
(**A**) *E. coli* culture containing pBHA bacteria proliferated in LB medium containing ampicillin. (**B**) *E. coli* growth curve in an ampicillin-containing medium.

In the current study, the extracted LegH underwent a series of experiments to determine the safety level regarding toxic and allergic ingredients. *E. coli* culture confirmed that pBHA-containing bacteria proliferated in LB medium containing ampicillin (Figure 5A). From the results of culturing *P. pastoris* with and without LegH sequences in YPD medium, a color difference could be observed when observed with the naked eye.

Furthermore, based on the OD results, bacteria containing pBHA were grown in a medium containing ampicillin. Bacteria with a dilution factor of 10 were confirmed to grow 1 h after inoculation, and observable growth of bacteria with a dilution factor of 10^9^ started after 18 h. Thus, it was confirmed that the growth rate was slow in samples with a high dilution factor because of the low number of initial bacteria. *P. pastoris* containing pJAN was able to grow in YPD medium containing ampicillin because of ampicillin resistance conferred by the pJAN plasmid (Figure 5B).

As shown in Table 1, the LegH sequence used did not match the sequences of allergens and toxins that cause allergies. Additionally, 11 *P. pastoris* proteins with allergen and 13 proteins with toxic protein homology showed similarity to the protein of *Saccharomyces cerevisiae*, a microorganism previously used for food. In *P. pastoris*, LegH digestion occurs by the proteolytic enzyme pepsin, at pH 2, similar to that of the stomach [41]. Heme has a long history of safe consumption, while soy LegH is a novel food ingredient. Therefore, soy LegH was subjected to rigorous safety testing, including tests for allergenicity, mutagenicity, chromosome damage, and toxicity [32]. In addition to being rapidly digested by pepsin, soy LegH does not share any meaningful similarity to known allergens or toxins [41,65]. Furthermore, LegH produced by *P. pastoris* and a 28-day trial on animal subjects indicated that no mortality was associated with LegH administration. This further clarified that there was no LegH-associated adverse clinical evidence, including metabolic, ophthalmological, or histopathological alterations [32]. Therefore, the consumption of LegH using *P. pastoris* as a food ingredient has no adverse effects, particularly allergenic and toxic reactions.

Additionally, Impossible™ Foods incorporated soy LegH protein using the yeast *P. pastoris*. LegH generated from the nodules of soy plants acts as a plant-based heme molecule. Moreover, LegH has tremendous Fe-binding capabilities, with regulatory functions in oxygen tension and diffusion. The broad-range resemblances of LegH and hemoglobin are red coloration, Fe-binding capability, and flavor [32]. Impossible™ Foods transferred the soy LegH gene into yeast to produce large quantities of LegH protein as sustainably as possible. Production of this ingredient by yeast fermentation has a smaller environmental footprint than digging up soybean root nodules and extracting the protein; however, it is identical to the LegH protein found in such root nodules [41]

In addition, the detection and purification of modified LegH were extracted from soybean root nodules. Nodule extracts were first chromatographed on hydroxylapatite at pH 6.8. Over 98% of the peptidases bound to the column and the LegH were recovered in the wash fraction, and subsequent ion-exchange chromatography of the wash fraction at pH 8.0 yielded several fractions [66]. The previous supporting literature indicated a successful extraction of LegH from soy roots [67,68,69].

#### 3.3.2. SDS-PAGE

**Figure 6:**
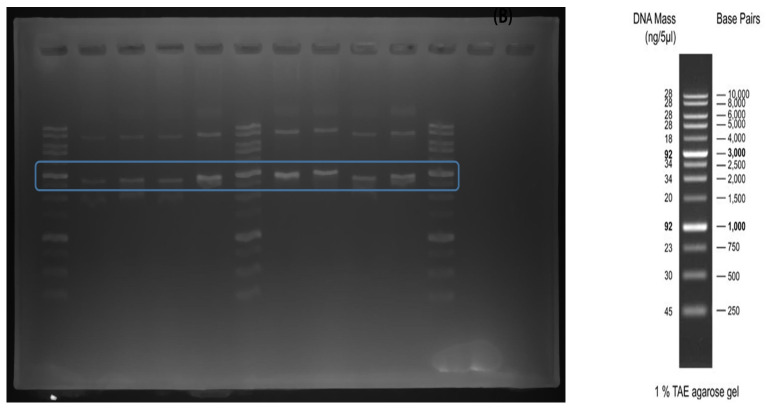
Electrophoresis results & 1 kb ladder. The targeted LegH sequence potentially identified at 2500 bp (Blue line).

The results for the extracted pBHA were confirmed by electrophoresis. The basic pBHA sequence size was 1987 bp, the LegH-producing sequence was 528 bp, and when the LegH sequence was inserted into pBHA, the size was 2515 bp. The corresponding size was inferred using the 1 kb reference ladder, the sample could be identified at approximately 2500 bp (Figure 6).

### 3.4. Color Measurements Pigments

The color coordinates of the extracted pigments for the PBM are listed in Table 2 and pigment weight and concentration are demonstrated in Table 3. Myoglobin, as a control, revealed color coordinates with the L* (6.84), a* (10.95), and b* (6.23) values. Similarly, the Fe-chlorophyll sample showed the highest L* value (22.59), whereas a* exhibited 10.60 and b* value exhibited 26.29; these values were all significantly different (*p* < 0.05) from the control. Based on the color indices, the L* value of anthocyanin was 3.90 and the a* value was 5.85, which was the fourth highest value, except for the control. However, LegH showed no significant difference with the control.

The extraction and generation of natural pigments for PBM is a challenging task, despite the availability of different renewable sources and various approaches [70]. A possible reason for this difficulty could be that the optimal pH of the colorant is dissimilar. This limitation of natural colorants is more likely to be overcome by adding acidulants, such as acetic acid, citric acid, or lactic acid [13].

**Figure 7:**
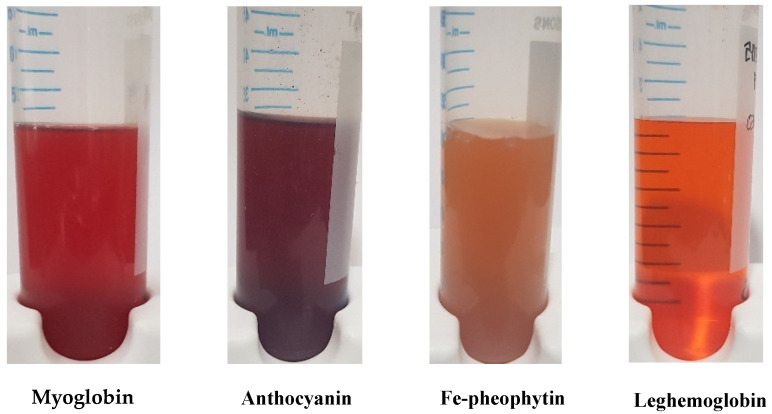
Pigment samples 30 mL in a 50 mL conical tube (Φ 3 cm) each.

In the current study, myoglobin, Fe-chlorophyll, and anthocyanin extracts were optimized to the level used in PBM products (Figure 7). For instance, some companies, such as Beyond Meat and Light life, use beet juice or powder to imitate a “bleeding” state for their products, whereas Impossible™ Foods uses soy LegH to confer a red meat-like appearance in its burger products [3]. Various food colorants from natural sources have been incorporated into various foods, such as anthocyanins [13,20,21,22], chlorophyll derivatives [29,30,62,63], and LegH [32,41]. The incorporation of natural colorants into patties were recently reported: the authors synergistically integrated beetroot pigments, laccase, and pectin to enhance the color coordinates of PBM patties [6]. Similarly, decolonization and detoxication of plant-based proteins were reported [11].

Previously, our team optimized and extracted two natural colorants (red yeast rice and lactoferrin), and thereafter synergistically incorporated these colorants into PBM analog patties; this ultimately resulted in the production of PBM analog patties that resembled real meat in many aspects [19].

### 3.5. Absorbance Level

**Figure 8:**
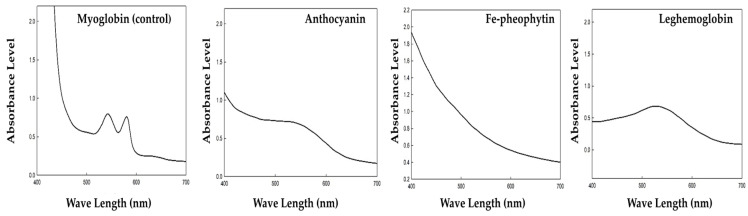
Absorbance level of pigment samples.

The concentrations of anthocyanin, chlorophyll and LegH extracted were determined at an absorbance of 0.7 at 535 nm. The absorbance at 0.7 was measured at 0.54 g of anthocyanin, 1.00 g of Fe-chlorophyll, and 1.76 g of LegH. The absorbance measurement spectrum and pigment weight with the concentration per colorant are shown in Figure 8. In addition, ideal extraction conditions were determined for each particular dye by determining the maximum absorbance level or optical density value at a precise absorbance wavelength using a UV-visible absorbance spectrophotometer [33]. By measuring the absorbance of the samples, it was confirmed that the absorbance increased from 400 nm to 535 nm and then decreased after the maximum value of 535 nm. Anthocyanins and Fe-chlorophyll showed a decreasing pattern after a 400 nm wavelength. These results correspond to those previously described by Nelson and Ferruzzi [29], and Furuya, Inoue, and Shirai [59]: both studies confirmed the effective synthesis of Fe-pheophytin derivatives in crude spinach extracts. Moreover, He and Giusti [22] describe in detail the incorporation of anthocyanins as natural colorants into food additives, and their absorbance and toxicity levels. The absorbance of the level of various natural pigments has been reported previously by various authors [18,71,72].

## 4. Conclusions

Natural pigments are exceptional sources of bioactive combinations that are considered harmless for human consumption. In this study, anthocyanins extracted from black beans, Fe-chlorophyll derived from spinach, and LegH were produced from microbial culture after the recombination and insertion of a modified plasmid. The effectiveness of anthocyanin extraction is the result of a combination of the solvent used and the applied technique. The chromatographic results reveal successful indications of anthocyanin-related compounds in the extracted sample from beans. Through microbial culture, the leghemoglobin was successfully synthesized by plasmid insertion and the sequence indicated negligible homology to toxic agents. The chlorophylls extracted from spinach and their synthesized derivatives including Fe-pheophytins were characterized by the ion exchange method. Furthermore, extraction technologies should be optimized to provide environmentally feasible and low-cost colorants from traditional and novel sources. The use of natural colorants in food systems is still limited because of technological issues. Therefore, further investigations are needed to determine how to stabilize these colorants in a diverse range of pH and temperatures for use in future food systems.

## Figures and Tables

**Table 1:** Sequences of pBHA, LegH, and pBHA + LegH.

	Treatment
	Sequence
**pBHA (1987 bp)**	GAATTCAGCCAGCAAGACAGCGATATCACCTGTAAGTCGGAC GAATTCGGCGCTCTTCCGCTTCCTCGCTCACTGACTCGCTGCGCTCGGTCGTTCGGCTGCGGCGAGCGGTATCAGCTCACTCAAAG GCGGTAATACGGTTATCCACAGAATCAGGGGATAACGCAGGAAAGAACATGTGAG CAAAAGGCCAGCAAAAGGCCAGGAACCGTAAAAAGGCCGCGTTGCTGGCGTTTTTCCATAGGCTCCGCCCCCCTGACGAGCATC ACAAAAATCGACGCTCAAGTCAGAGGTGGCGAAACCCGACAGGACTATAAAGATAC CAGGCGTTTCCCCCTGGAAGCTCCCTCGTGCGCTCTCCTGTTCCGACCCTGCCGCTTACCGGATACCTGTCCGCCTTTCTCCCTTCG GGAAGCGTGGCGCTTTCTCATAGCTCACGCTGTAGGTATCTCAGTTCGGTG TAGGTCGTTCGCTCCAAGCTGGGCTGTGTGCACGAACCCCCCGTTCAGCCCGACCGCTGCGCCTTATCCGGTAACTATCGTCTTGA GTCCAACCCGGTAAGACACGACTTATCGCCACTGGCAGCAGCCACTGGTAACAGGATTAG CAGAGCGAGGTATGTAGGCGGTGCTACAGAGTTCTTGAAGTGGTGGCCTAACTACGGCTACACTAGAAGAACAGTATTTGGTATC TGCGCTCTGCTGAAGCCAGTTACCTTCGGAAAAAGAGTTGGTAGCTCTTGATCCGG CAAACAAACCACCGCTGGTAGCGGTGGTTTTTTTGTTTGCAAGCAGCAGATTACGCGCAGAAAAAAAGGATCTCAAGAAGATCCT TTGATCTTTTCTACGGGGTCTGACGCTCAGTGGAACGAAAACTCACGTTAAGGGATTTT GGTCATGAGATTATCAAAAAGGATCTTCACCTAGATCCTTTTAAATTAAAAATGAAGTTTTAAATCAATCTAAAGTATGAGTAAA CTTGGTCTGACAGTTACCAATGCTTAATCAGTGAGGCACCTATCTCAGCGATCTGTC TATTTCGTTCATCCATAGTTGCCTGACTCCCCGTCGTGTAGATAACTACGATACGGGAGGGCTTACCATCTGGCCCCAGTGCTGCA ATGATACCGCGAGACCCACGCTCACCGGCTCCAGATTTATCAGCAATAAACCAGCCAGCCG GAAGGGCCGAGCGCAGAAGTGGTCCTGCAACTTTATCCGCCTCCATCCAGTCTATTAATTGTTGCCGGGAAGCTAGAGTAAGTAG TTCGCCAGTTAATAGTTTGCGCAACGTTGTTGCCATTGCTACAGGCATCGTGGTGTCAC GCTCGTCGTTTGGTATGGCTTCATTCAGCTCCGGTTCCCAACGATCAAGGCGAGTTACATGATCCCCCATGTTGTGCAAAAAAGCG GTTAGCTCCTTCGGTCCTCCGATCGTTGTCAGAAGTAAGTTGGCCGCAGTGTTATCAC TCATGGTTATGGCAGCACTGCATAATTCTCTTACTGTCATGCCATCCGTAAGATGCTTTTCTGTGACTGGTGAGTACTCAACCAAGT CATTCTGAGAATAGTGTATGCGGCGACCGAGTTGCTCTTGCCCGGCGTCAATACGGGATAA TACCGCGCCACATAGCAGAACTTTAAAAGTGCTCATCATTGGAAAACGTTCTTCGGGGCGAAAACTCTCAAGGATCTTACCGCTG TTGAGATCCAGTTCGATGTAACCCACTCGTGCACCCAACTGATCTTCAG CATCTTTTACTTTCACCAGCGTTTCTGGGTGAGCAAAAACAGGAAGGCAAAATGCCGCAAAAAAGGGAATAAGGGCGACACGGA AATGTTGAATACTCATACTCTTCCTTTTTCAATATTATTGAA GCATTTATCAGGGTTATTGTCTCATGAGCGGATACATATTTGAATGTATTTAGAAAAATAAACAAATAGGGGTTCCGCGCACATTT CCCCGAAAAGTGCCACGTGA
**LegH (528 bp)**	CCATGGAAGCTTCATCATCATCATCATCACGCGGCCGCG GATCCGAATTCGTCGACAGATCTAGGCCTATGGGTGCTTTTACTGAAAAGCAAGAGGCTTTGGTTTCTTCTTCTTTTGAGGCTTTTA AGGCTAACATTCCACAATACTCTGTTGTTTTCTATACTTCTATTTTGGA GAAGGCTCCTGCTGCTAAAGATTTGTTTTCTTTCTTGTCTAACGGTTTGATCCATCTAATCCTAAGTTGACTGGTCATGCTGAAAAA TTGTTTGGTTTGGTTAGAGATTCTGCTGGTCAATTGAAGGCTAATGGTACTGTTGTT GCTGATGCTGCTTTGGGTTCTATTCACGCTCAAAAGGCTATTACTGATCCACAATTCGTTGTTGTTAAGGAGGCTTTGTTGAAAACT ATTAAGGAGGCTGTTGGAGATAAATGGTCTGATGAATTGTCCTCTGCTTGGGAAGTT GCTTACGATGAATTGGCTGCTGCTATTAAGAAGGCTTTTCACCACCACCACCACCACGCTGAGC
**pBHA** **+ LegH** **(2515 bp)**	GAATTCAGCCAGCAAGACAGCGATCCATGGAAGCTTCATCATCATCATCATCAC GCGGCCGCGGATCCGAATTCGTCGACAGATCTAGGCCTATGGGTGCTTTTACTGAAAAGCAAGAGGCTTTGGTTTCTTCTTCTTTTGAGG CTTTTAAGGCTAACATTCCACAATACTCTGTTGTTTTCTATACTTCTATTTTGGA GAAGGCTCCTGCTGCTAAAGATTTGTTTTCTTTCTTGTCTAACGGTGTTGATCCATCTAATCCTAAGTTGACTGGTCATGCTGAAAAATTG TTTGGTTTGGTTAGAGATTCTGCTGGTCAATTGAAGGCTAATGGTACTGTTGTT GCTGATGCTGCTTTGGGTTCTATTCACGCTCAAAAGGCTATTACTGATCCACAATTCGTTGTTGTTAAGGAGGCTTTGTTGAAAACTATTA AGGAGGCTGTTGGAGATAAATGGTCTGATGAATTGTCCTCTGCTTGGGAAGTTGCTTAC GATGAATTGGCTGCTGCTATTAAGAAGGCTTTTCACCACCACCACCACCACGCTGA GCATCACCTGTAAGTCGGACGAATTCGGCGCTCTTCCGCTTCCTCGCTCAC TGACTCGCTGCGCTCGGTCGTTCGGCTGCGGCGAGCGGTATCAGCTCACTCAAAGGCGGTAATACGGTTATCCACAGAATCAGGGGATA ACGCAGGAAAGAACATGTGAGCAAAAGGCCAGCAAAAGGCCAGGAACCG TAAAAAGGCCGCGTTGCTGGCGTTTTTCCATAGGCTCCGCCCCCCTGACGAGCATCACAAAAATCGACGCTCAAGTCAGAGGTGGCGAA ACCCGACAGGACTATAAAGATAC CAGGCGTCTCCCTCGTGCGCTCTCCTGTTCCGACCCTGCCGCTTACCGGATACCTGTCCGCCTTTCTCCCTTCGGGAAGCGTGGCGCTTTC TCATAGCTCACGCTGTAGGTATCTCAGTTCGGTGTAGGTCGTTCGCTCCAA GCTGGGCTGTGTGCACGAACCCCCCGTTCAGCCCGACCGCTGCGCCTTATCCGGTAACTATCGTCTTGAGTCCAACCCGGTAAGACACGA CTTATCGCCACTGGCAGCAGCCACTGGTAACAGGATTAG CAGAGCGAGGTATGTAGGCGGTGCTACAGAGTTCTTGAAGTGGTGGCCTAACTACGGCTACACTAGAAGAACAGTATTTGGTATCTGCG CTCTGCTGAAGCCAGTTACCTTCGGAAAAAGAGTTGGTAGCTCTTGATCCGGCAAACAAAC CACCGCTGGTAGCGGTGGTTTTTTTGTTTGCAAGCAGCAGATTACGCGCAGAAAAAAAGGATCTCAAGAAGATCCTTTGATCTTTTCTAC GGGGTCTGACGCTCAGTGGAACGAAAACTCACGTTAAGGGATTTTGGTCATGAGAT TATCAAAAAGGATCTTCACCTAGATCCTTTTAAATTAAAAATGAAGTTTTAAATCAATCTAAAGTATATATGAGTAAACTTGGTCTGACA GTTACCAATGCTTAATCAGTGAGGCACCTATCTCAGCGATCTGTCTATTTCGTTCATCCA TAGTTGCCTGACTCCCCGTCGTGTAGATAACTACGATACGGGAGGGCTTACCATC

**Table 2:** Pigment samples color coordinates.

Color Indices	Myoglobin	Fe-pheophytin	Anthocyanin	Leghemoglobin
Lightness (L*)	6.84 ± 0.01 ^c^	22.59 ± 0.05 ^a^	3.90 ± 0.01 ^d^	6.82 ± 0.01 ^c^
Redness (a*)	10.95 ± 0.01 ^b^	10.60 ± 0.03 ^c^	5.82 ± 0.01 ^d^	9.89 ± 0.01 ^b^
Yellowness (b*)	6.23 ± 0.06 ^c^	26.29 ± 0.04 ^b^	1.89 ± 0.02 ^d^	6.50 ± 0.05 ^c^

The results are shown as mean ± standard deviation (*n* = 3). a, b, c, d Means with the different letters are significantly different (*p* < 0.05).

**Table 3:** Pigment samples weight and concentration.

Sample	Concentrations (%)	Pigments Weight (g)
Leghaemoglobin	0.37	0.11
Anthocyanin	0.54	0.18
Fe-chlorophyll	1.00	0.30
Myoglobin (control)	1.76	0.53

Pigment weight and concentration per colorant (30 mL).

## Data Availability

The data presented in this study are available on request from the corresponding author.

## References

[1] Bakhsh A., Lee S.-J., Lee E.-Y., Hwang Y.-H., Joo S.-T. (2021). Traditional plant-based meat alternatives, current, and future perspective: A review. J. Agric. Life Sci..

[2] Bakhsh A., Lee E.-Y., Ncho C.M., Kim C.-J., Son Y.-M., Hwang Y.-H., Joo S.-T. (2022). Quality Characteristics of Meat Analogs through the Incorporation of Textured Vegetable Protein: A Systematic Review. Foods.

[3] Bohrer B.M. (2019). An investigation of the formulation and nutritional composition of modern meat analogue products. Food Sci. Hum. Wellness.

[4] Ahmad M., Hirz M., Pichler H., Schwab H. (2014). Protein expression in Pichia pastoris: Recent achievements and perspectives for heterologous protein production. Appl. Microbiol. Biotechnol..

[5] Kumar P., Chatli M., Mehta N., Singh P., Malav O., Verma A.K. (2017). Meat analogues: Health promising sustainable meat substitutes. Crit. Rev. Food Sci. Nutr..

[6] Sakai K., Sato Y., Okada M., Yamaguchi S. (2022). Synergistic effects of laccase and pectin on the color changes and functional properties of meat analogs containing beet red pigment. Sci. Rep..

[7] Sakai K., Sato Y., Okada M., Yamaguchi S. (2021). Improved functional properties of meat analogs by laccase catalyzed protein and pectin crosslinks. Sci. Rep..

[8] Godfray H.C.J., Aveyard P., Garnett T., Hall J.W., Key T.J., Lorimer J., Pierrehumbert R.T., Scarborough P., Springmann M., Jebb S.A. (2018). Meat consumption, health, and the environment. Science.

[9] Fehér A., Gazdecki M., Véha M., Szakály M., Szakály Z. (2020). A Comprehensive Review of the Benefits of and the Barriers to the Switch to a Plant-Based Diet. Sustainability.

[10] Sakai K., Sato Y., Okada M., Yamaguchi S. (2022). Cyclodextrins produced by cyclodextrin glucanotransferase mask beany off-flavors in plant-based meat analogs. PLoS ONE.

[11] Sakai K., Okada M., Yamaguchi S. (2022). Decolorization and detoxication of plant-based proteins using hydrogen peroxide and catalase. Sci. Rep..

[12] He J., Evans N.M., Liu H., Shao S. (2020). A review of research on plant-based meat alternatives: Driving forces, history, manufacturing, and consumer attitudes. Compr. Rev. Food Sci. Food Saf..

[13] Luzardo-Ocampo I., Ramírez-Jiménez A.K., Yañez J., Mojica L., Luna-Vital D.A. (2021). Technological applications of natural colorants in food systems: A review. Foods.

[14] Manzoor M., Singh J., Gani A., Noor N. (2021). Valorization of natural colors as health-promoting bioactive compounds: Phytochemical profile, extraction techniques, and pharmacological perspectives. Food Chem..

[15] Albuquerque B.R., Pinela J., Barros L., Oliveira M.B.P., Ferreira I.C. (2020). Anthocyanin-rich extract of jabuticaba epicarp as a natural colorant: Optimization of heat-and ultrasound-assisted extractions and application in a bakery product. Food Chem..

[16] Cortez R., Luna-Vital D.A., Margulis D., Gonzalez de Mejia E. (2017). Natural pigments: Stabilization methods of anthocyanins for food applications. Compr. Rev. Food Sci. Food Saf..

[17] Selig M.J., Gamaleldin S., Celli G.B., Marchuk M.A., Smilgies D.-M., Abbaspourrad A. (2020). The stabilization of food grade copper-chlorophyllin in low pH solutions through association with anionic polysaccharides. Food Hydrocoll..

[18] Ghosh S., Sarkar T., Das A., Chakraborty R. (2022). Natural colorants from plant pigments and their encapsulation: An emerging window for the food industry. LWT.

[19] Bakhsh A., Lee E.-Y., Bakry A.M., Rathnayake D., Son Y.-M., Kim S.-W., Hwang Y.-H., Joo S.-T. (2022). Synergistic effect of lactoferrin and red yeast rice on the quality characteristics of novel plant-based meat analog patties. LWT.

[20] Takeoka G., Dao L. (2002). Anthocyanins In Methods of Analysis for Functional Foods and Nutraceuticals.

[21] Wrolstad R.E., Culver C.A. (2012). Alternatives to those artificial FD&C food colorants. Annu. Rev. Food Sci. Technol..

[22] He J., Giusti M.M. (2010). Anthocyanins: Natural colorants with health-promoting properties. Annu. Rev. Food Sci. Technol..

[23] Wu X., Beecher G.R., Holden J.M., Haytowitz D.B., Gebhardt S.E., Prior R.L. (2006). Concentrations of anthocyanins in common foods in the United States and estimation of normal consumption. J. Agric. Food Chem..

[24] FAO Joint, World Health Organization, WHO Expert Committee on Food Additives (2017). Evaluation of Certain Contaminants in Food: Eighty-Third Report of the Joint FAO/WHO Expert Committee on Food Additives.

[25] Garzón G., Wrolstad R. (2001). The stability of pelargonidin-based anthocyanins at varying water activity. Food Chem..

[26] Amr A., Al-Tamimi E. (2007). Stability of the crude extracts of Ranunculus asiaticus anthocyanins and their use as food colourants. Int. J. Food Sci. Technol..

[27] Ghosh D., Konishi T. (2007). Anthocyanins and anthocyanin-rich extracts: Role in diabetes and eye function. Asia Pac. J. Clin. Nutr..

[28] Tsuda T. (2008). Regulation of adipocyte function by anthocyanins; possibility of preventing the metabolic syndrome. J. Agric. Food Chem..

[29] Nelson R., Ferruzzi M. (2008). Synthesis and Bioaccessibility of Fe-Pheophytin Derivatives from Crude Spinach Extract. J. Food Sci..

[30] Hsu C.-Y., Chao P.-Y., Hu S.-P., Yang C.-M. (2013). The antioxidant and free radical scavenging activities of chlorophylls and pheophytins. Food Nutr. Sci..

[31] No J., Yoon H., Park S., Yoo S.J., Shin M. (2016). Color stability of chlorophyll in young barley leaf. J. East Asian Soc. Diet. Life.

[32] Fraser R.Z., Shitut M., Agrawal P., Mendes O., Klapholz S. (2018). Safety evaluation of soy leghemoglobin protein preparation derived from Pichia pastoris, intended for use as a flavor catalyst in plant-based meat. Int. J. Toxicol..

[33] Prabhu K., Bhute A.S. (2012). Plant based natural dyes and mordants: A Review. J. Nat. Prod. Plant Resour..

[34] Pappas V.M., Athanasiadis V., Palaiogiannis D., Poulianiti K., Bozinou E., Lalas S.I., Makris D.P. (2021). Pressurized Liquid Extraction of Polyphenols and Anthocyanins from Saffron Processing Waste with Aqueous Organic Acid Solutions: Comparison with Stirred-Tank and Ultrasound-Assisted Techniques. Sustainability.

[35] Chávez-Santoscoy R.A., Lazo-Vélez M.A., Serna-Sáldivar S.O., Gutiérrez-Uribe J.A. (2016). Delivery of flavonoids and saponins from black bean (Phaseolus vulgaris) seed coats incorporated into whole wheat bread. Int. J. Mol. Sci..

[36] Heravi E.J., Aghdam H.H., Puig D. Classification of Foods Using Spatial Pyramid Convolutional Neural Network. Proceedings of the 19th International Conference of the Catalan Association for Artificial Intelligence.

[37] Moustafa Y.M., Morsi R.E. (2013). Ion exchange chromatography-An overview. Column Chromatogr..

[38] Tran L., Rathinasamy V.A., Beddoe T. (2022). Development of a loop-mediated isothermal amplification assay for detection of Austropeplea tomentosa from environmental water samples. Anim. Dis..

[39] Food and Drug Administration USA Impossible Foods, Inc.; Filing of Color Additive Petition. https://www.federalregister.gov/documents/2018/12/13/2018-26949/impossible-foods-inc-filing-of-color-additive-petition.

[40] De Boer A., Krul L., Fehr M., Geurts L., Kramer N., Urbieta M.T., Van Der Harst J., Van De Water B., Venema K., Schütte K. (2020). Animal-free strategies in food safety & nutrition: What are we waiting for? Part I: Food safety. Trends Food Sci. Technol..

[41] Jin Y., He X., Andoh-Kumi K., Fraser R.Z., Lu M., Goodman R.E. (2018). Evaluating potential risks of food allergy and toxicity of soy leghemoglobin expressed in Pichia pastoris. Mol. Nutr. Food Res..

[42] Bakhsh A., Lee S.-J., Lee E.-Y., Hwang Y.-H., Joo S.-T. (2021). Characteristics of Beef Patties Substituted by Different Levels of Textured Vegetable Protein and Taste Traits Assessed by Electronic Tongue System. Foods.

[43] Ismail I., Hwang Y.-H., Bakhsh A., Joo S.-T. (2019). The alternative approach of low temperature-long time cooking on bovine semitendinosus meat quality. Asian-Australas. J. Anim. Sci..

[44] Bakhsh A., Lee S.-J., Lee E.-Y., Sabikun N., Hwang Y.-H., Joo S.-T. (2021). A Novel Approach for Tuning the Physicochemical, Textural, and Sensory Characteristics of Plant-Based Meat Analogs with Different Levels of Methylcellulose Concentration. Foods.

[45] Fernández-López J.A., Angosto J.M., Giménez P.J., León G. (2013). Thermal stability of selected natural red extracts used as food colorants. Plant Foods Hum. Nutr..

[46] Sabikun N., Bakhsh A., Rahman M.S., Hwang Y.-H., Joo S.-T. (2021). Volatile and nonvolatile taste compounds and their correlation with umami and flavor characteristics of chicken nuggets added with milkfat and potato mash. Food Chem..

[47] Crozier A., Jaganath I.B., Clifford M.N. (2006). Phenols, polyphenols and tannins: An overview. Plant Second. Metab. Occur. Struct. Role Hum. Diet.

[48] Lianza M., Marincich L., Antognoni F. (2022). The Greening of Anthocyanins: Eco-Friendly Techniques for Their Recovery from Agri-Food By-Products. Antioxidants.

[49] Teixeira R.F., Benvenutti L., Burin V.M., Gomes T.M., Ferreira S.R.S., Zielinski A.A.F. (2021). An eco-friendly pressure liquid extraction method to recover anthocyanins from broken black bean hulls. Innov. Food Sci. Emerg. Technol..

[50] Pomar F., Novo M., Masa A. (2005). Varietal differences among the anthocyanin profiles of 50 red table grape cultivars studied by high performance liquid chromatography. J. Chromatogr. A..

[51] Ji Y., Fan Y., Liu K., Kong D., Lu J. (2015). Thermo activated persulfate oxidation of antibiotic sulfamethoxazole and structurally related compounds. Water Res..

[52] Khoo H.E., Azlan A., Tang S.T., Lim S.M. (2017). Anthocyanidins and anthocyanins: Colored pigments as food, pharmaceutical ingredients, and the potential health benefits. Food Nutr. Res..

[53] Bendokas V., Skemiene K., Trumbeckaite S., Stanys V., Passamonti S., Borutaite V., Liobikas J. (2020). Anthocyanins: From plant pigments to health benefits at mitochondrial level. Crit. Rev. Food Sci. Nutr..

[54] Nitteranon V., Kittiwongwattana C., Vuttipongchaikij S., Sakulkoo J., Srijakkoat M., Chokratin P., Harinasut P., Suputtitada S., Apisitwanich S. (2014). Evaluations of the mutagenicity of a pigment extract from bulb culture of *Hippeastrum reticulatum*. Food Chem. Toxicol..

[55] Berger J., Dillon J.-C. (2002). Control of iron deficiency in developing countries. Cah. D’études Et De Rech. Francoph./St..

[56] Brown S., Houghton J., Hendry G., Scheer H. (1991). Chlorophylls.

[57] Tonucci L.H., Von Elbe J.H. (1992). Kinetics of the formation of zinc complexes of chlorophyll derivatives. J. Agric. Food Chem..

[58] Nonomura Y., Yamaguchi M., Hara T., Furuya K., Yoshioka N., Inoue H. (1996). High-performance liquid chromatographic separation of iron (III) chlorophyllin. J. Chromatogr. A.

[59] Furuya K., Inoue H., Shirai T. (1987). Determination of pheophytinatoiron (III) chlorides by reversed phase high performance liquid chromatography. Anal. Sci..

[60] Britton G. (1983). The Biochemistry of Natural Pigments.

[61] Schwartz S., Von Elbe J. (1983). Kinetics of chlorophyll degradation to pyropheophytin in vegetables. J. Food Sci..

[62] Solymosi K., Mysliwa-Kurdziel B. (2017). Chlorophylls and their derivatives used in food industry and medicine. Mini Rev. Med. Chem..

[63] Kang Y.-R., Park J., Jung S.K., Chang Y.H. (2018). Synthesis, characterization, and functional properties of chlorophylls, pheophytins, and Zn-pheophytins. Food Chem..

[64] Von Elbe J., Schwartz S. (1996). Colorants. Food Chemistry.

[65] Reyes T.F., Chen Y., Fraser R.Z., Chan T., Li X. (2021). Assessment of the potential allergenicity and toxicity of Pichia proteins in a novel leghemoglobin preparation. Regul. Toxicol. Pharmacol..

[66] Jun H.-K., Sarath G., Wagner F.W. (1994). Detection and purification of modified leghemoglobins from soybean root nodules. Plant Sci..

[67] Anderson C., Jensen E.O., Llewellyn D.J., Dennis E.S., Peacock W.J. (1996). A new hemoglobin gene from soybean: A role for hemoglobin in all plants. Proc. Natl. Acad. Sci. USA.

[68] Kosmachevskaya O.V., Nasybullina E.I., Shumaev K.B., Topunov A.F. (2021). Expressed soybean leghemoglobin: Effect on *Escherichia coli* at oxidative and nitrosative stress. Molecules.

[69] Hargrove M.S., Barry J.K., Brucker E.A., Berry M.B., Phillips G.N., Olson J.S., Arredondo-Peter R., Dean J.M., Klucas R.V., Sarath G. (1997). Characterization of recombinant soybean leghemoglobin a and apolar distal histidine mutants. J. Mol. Biol..

[70] Bakhsh A., Lee S.-J., Lee E.-Y., Hwang Y.-H., Joo S.-T. (2021). Evaluation of rheological and sensory characteristics of plant-based meat analog with comparison to beef and pork. Food Sci. Anim. Resour..

[71] Basuki, Suyitno, Kristiawan B. (2017). Absorbance and electrochemical properties of natural indigo dye. AIP Conference Proceedings, Proceedings of the The 3rd International Conference on Industrial, Mechanical, Electrical, and Chemical Engineering, Surakarta, Indonesia, 13–14 September 2017.

[72] Singhee D., Sarkar A. (2022). Colorimetric Measurement and Functional Analysis of Selective Natural Colorants Applicable for Food and Textile Products. Colorimetry.

